# The psychosocial impact of caregiving on the family caregivers of chronically ill AIDS and/or HIV patients in home-based care: A qualitative study in Zimbabwe

**DOI:** 10.4102/sajhivmed.v18i1.718

**Published:** 2017-12-05

**Authors:** Claire van Deventer, Anne Wright

**Affiliations:** 1 Department of Family Medicine, University of the Witwatersrand, South Africa

## Abstract

**Background:**

The family caregiver has a pivotal role to play in the management of the chronically ill HIV and/or AIDS patients. The wellbeing of caregivers is therefore crucial because impairment of their physical or mental health could impact negatively on the management of their HIV-positive family member. The purpose of this qualitative study was to explore the psychosocial impact of caregiving on the family caregiver of the chronically ill HIV and/or AIDS patients in home-based care.

**Method:**

Unstructured interviews were conducted with 11 caregivers recruited at an adult HIV clinic at United Bulawayo Hospitals, Bulawayo, Zimbabwe. Relevant demographic information was collected from each participant. The interviews were then transcribed and analysed.

**Results:**

Caregivers’ biggest challenge was meeting care costs such as food, transport and medical costs. Certain conditions relating to the care-recipient’s health and family issues, such as abandonment of the ill patient as well as that of orphans, added to the burden of care. Carers also had to deal with their own health and physical problems. All the above resulted in a spectrum of emotions such as helplessness, sadness, anxiety and anger. Despite this, caregivers also reported on the positive aspects regarding their caregiving role.

**Conclusion:**

There were both negative and positive psychosocial experiences by caregivers of HIV and/or AIDS patients. The study highlighted practical areas where support could be provided.

## Introduction

The impact of caregiving on the wellbeing of carers has been studied in different communities across the world.^1,2,3,4,5,6^ The overall results revealed that these caregivers suffer from significant psychosocial problems. The problems encountered included depression, anxiety, loneliness, anger, fear, stigmatisation and economic difficulties. The extent of caregiving in these studies included assistance with activities of daily living (ADL), management of the disease and medications, as well as emotional and financial support.

In a Botswana study,^1^ older women complained of exhaustion because they were often caring for multiple family members. Their physical care for the ill patient as well as the general household chores like fetching firewood and water, cooking and cleaning resulted in neglect of their own health. The stigma of HIV extended to the carers and exacerbated the isolation they felt.

A study which was performed exclusively with elderly caregivers in rural Uganda^6^ showed similar results. Additional factors in this group were their fear of contracting the illness through the caring process and a sense of futility. In rural Namibia,^7^ diaries were kept by participants. These revealed, amongst others, that over time tensions arose between the caregiver and patient as well as in the household in general. This resulted in the carer becoming insensitive to and withdrawing from the patient. In contrast to these problem-saturated accounts, a qualitative study in Malawi^8^ reported that nine out of 15 caregivers experienced no problems in their caregiver roles. Many of these patients died within a few months, and this may explain why caregiver burnout did not occur.

Most carers in these African studies were women from rural, impoverished communities. However, in the United States and Australia,^9,10^ where palliative care was more often given to gay men, it was found that male caregivers were more prevalent because of patient choice.

Zimbabwe, where the study was conducted, has been significantly affected by the AIDS pandemic. This country reported the 5th highest HIV prevalence in sub-Saharan Africa in 2015, at 15%.^11^ The adult prevalence has however almost halved from 29% in 1998.^11^ The number of adults receiving antiretroviral therapy (ART) in 2014 was 89.7%.^11^ These indicators have all improved significantly in the last 10 years. There has been a strong commitment from government with various financial and political initiatives being implemented. These have, however, been countered by the general economic deterioration in the country. Healthcare, as one of many sectors, has been affected by the financial situation, which led to the care of the chronically ill devolving largely to the family. In many other countries, home-based care is an integral part of the management of these patients. This is because of the additional human and economic burdens, protracted hospital admissions and the sheer numbers of very ill patients. No similar studies regarding carers of HIV and/or AIDS patients were found relating to Zimbabwean citizens, which provided an opportunity for the researcher to contribute additional information.

The study was carried out because encounters with caregivers in clinical practice often revealed feelings of despair. The researcher also observed impatience or anger between partners and also that elderly relatives were involved in many instances in fulfilling the role of carer. The aim of the research was to study the impact of caregiving on the psychosocial health of family caregivers of HIV and/or AIDS patients in home-based care, describing their demographic profile and their perspectives on the effects this role was having on them.

## Methods

### Design

A qualitative study was performed because ‘human emotions are difficult to quantify; qualitative research seems to be more effective than quantitative research for investigating these emotional responses’.^12^ In particular, individual interviews were expected to contribute to this understanding regarding the topic and because of the site of the study, where many carers accompanied patients for their medial visits, this was considered to be a practically achievable method.

### Study site

The study was conducted at the Opportunistic Infection (OI) Clinic at United Bulawayo Hospitals (UBH) in Bulawayo, Zimbabwe. The clinic was dedicated to managing HIV patients only.

### Study population

Adult caregivers with any type of relationship to home-based chronically ill, adult HIV-positive patients who attended the OI Clinic at UBH.

### Sampling

Purposive sampling was performed, related to any accompanying caregivers at the clinic in the study period.

Inclusion criteria for participating in the study were the following: adults over 18 years, caregiving performed by these caregivers in the community, assistance with ADL as opposed to simple visits, a period of caregiving for longer than two weeks, residing in Bulawayo for accessibility and speaking English or Shona fluently. Child and adolescent carers were not included as they are in the minority and are faced with a different set of challenges.

A total of 14 participants were recruited, but 11 were interviewed as three were not available when the interviews were conducted. The sample size was determined by saturation where the researcher found no new information emerging.

### Measuring tools and data collection

Interviews took place at the caregiver’s home or the researcher’s office. Informed consent was obtained with separate consent for the audiotaping. Demographic information included age and gender of carer and patients, employment status, relationship of the carer with the patient, HIV status of the carer, duration of caregiving and the carer’s Karnofsky score.^13^ This latter scale measures the incapacity of the patient, which influences the degree of caregiving required. After completion of the questionnaire, the participant was invited to share his or her experience of the caregiving process by the use of an unstructured one-on-one interview. The key introductory question was: ‘How are you finding the experience of taking care of your ill relative?’ Additional questions were used only for the clarification of specific points as supported by qualitative research techniques.^14,15,16^ The interviewer employed the techniques of clarification, reflection and summarising in order to ascertain that the researcher had understood the participant’s full perspective and to encourage the flow of conversation between the interviewer and interviewee. The interview was recorded on audiotape, and some important points were captured in field notes in order to highlight issues such as non-verbal communication. The interviews were conducted exclusively by the researcher either in Shona or English or a combination of both and lasted an average of an hour. Each interview was then transcribed by the researcher on the same day to allow for better recall of the interview and other events surrounding it.

### Bias

The presence of the tape recorder may have intimidated some of the participants and could have affected the quality of the information shared. However, this did not seem to be a problem as none of the participants seemed to be paying any attention to the tape recorder.

To pre-empt any preconceived ideas and opinions from the researcher, her own ideas regarding the research topic were explored by a qualitative research trainer before the research commenced.

Language limitations may have excluded the opinions of relevant caregivers. An attempt was made to contact the participants after the analysis of the data, to validate the findings. Only five participants were able to do so as three had died and three were uncontactable.

It is acknowledged that during the analysis of qualitative research data, the researcher’s bias may play a role in the selection of quotes and the presentation thereof. Chenail makes it clear that ‘Data from qualitative research are … rarely if ever conclusive. Nevertheless the analysis process should be highly deliberate and systematic’.^17^ For this reason, triangulation was planned to confirm and validate the findings of the study. As indicated above, less than half the participants could be contacted for this step, which contributes to the possible bias.

### Analysis

Taped interviews were transcribed as soon after they were conducted as possible. The key to qualitative data analysis is to familiarise or ‘immerse’ oneself in the information contained in the transcripts.^18^ As the transcripts of interviews were analysed, common themes emerged from the verbatim quotes of the interviewees and these were highlighted in different colours. These were then further analysed to create subthemes. The cut and paste method was subsequently used to move quotes to particular themes.

### Limitations

The obvious limitations are those of any qualitative study in that the findings are not easily transferable to another population because of the sample size and non-random sampling. Analysis is also very dependent on the researcher’s skill and inherent subjective views^19^ as was mentioned earlier. Triangulation of observer, theory and method^14^ would have strengthened the research, but this was not possible logistically.

## Ethical consideration

Permission to conduct this study was obtained from the Medical Research Council of Zimbabwe, the Ethics Committee of the University of Zimbabwe and the Research and the Ethics Committee of the University of the Witwatersrand. All participants received an information sheet emphasising that the study was entirely voluntary, confidentiality was protected and tapes and transcripts would be kept in safekeeping by the researcher. Written consent was obtained from each participant. Counsellors at the OI Clinic were available for assistance in the event of emotional distress related to the interviews.

## Results

### Demographics

The caregivers’ demographics are presented in Table 1, with 10 of the 11 being women and seven of these over the age of 50. There were two spousal dyads. Nine carers did not know their own HIV status. The remaining two were HIV-positive themselves. Most of the carers interviewed had taken responsibility for one patient each, mostly direct relatives or spouses. The Karnofsky score was more than 50 in only three patients, indicating a moderately heavy carer load for most of the caregivers as a Karnofsky score of 50 indicates ‘moderately disabled; dependent. Requires considerable assistance and frequent care’.^13^ Lower scores indicate increasing disability and dependence.

**TABLE 1:** Demographic data of participants.

Participant number	Age	Gender	No of patients being cared for	Employment	Duration in care	Karnofsky score	Relationship with patients
1	69	F	4	Unemployed	3 weeks	30	Grandmother-in-law
2	44	F	1	Employed	3 weeks	70	Sister
3	56	F	1	Unemployed	1 year	40	Mother
4	59	F	1	Employed	3 months	60	Mother
5	36	M	1	Employed	1 month	50	Husband
6	34	F	1	Unemployed	3 months	50	Wife
7	58	F	1	Employed	3 years	50	Mother
8	58	F	1	Unemployed	7 months	40	Mother
9	40	F	3	Employed	8 months	50	Sister
10	64	F	1	Unemployed	4 months	60	Aunt
11	50	F	1	Employed	8 months	40	Mother

### Themes

The seven main themes identified were the following: financial issues, caregivers’ physical/health problems, patients’ physical/health problems, religious aspects, family issues, emotional impact and an appraisal of the caregiver role.

Given the financial challenges facing Zimbabwe, it was not surprising that financial difficulties were raised by all but one of the respondents.

These were because of several factors most of which were expressed by the majority of participants:

I do not have food or soap for washing her soiled linen. [P4, Female, 59]

and:

Isn’t it when the linen gets washed every day, it becomes old very quickly? [P4, Female, 59]

Sometimes she can choose food, whatever, and maybe at that I would have bought food that is adequate for the family but then she can say she doesn’t want it, she wants something else when I don’t even have a cent. [P9, Female, 40]

So when the day we have to take her to the hospital comes and she is not able to walk…The money for the fuel is what’s hard to find, they charge a lot. [P8, Female, 58]

So it is a problem not to have an income when you are caring for someone who is ill because how do you help them and yourself? [P3, Female, 56]

Additional burdens were where four of the caregivers were looking after the ill person’s children or orphans, some of whom were also ill. Medical needs were also mentioned by three carers as part of the financial problem:

Yes the drugs are so expensive and sometimes I can’t afford some of the drugs the doctor says we should buy. [P9, Female, 40]

Other financial issues that were mentioned related to the high inflation, lack of employment and the fact that household goods were being sold to make ends meet.

#### Caregivers’ health/physical issues

Most of the carers were older women with their own health problems and physical limitations. One carer acknowledged the limitations of her age:

…when one is as ill as she is, it is difficult to lift her, I am old, am very old. [P1, Female, 69]

and another her physical exhaustion:

…even during the day I always find that I’m always asleep, I sleep, myself always I sleep. I sit down, I’m asleep, at night I sleep. [P4, Female, 59]

There were also psychosomatic symptoms mentioned:

I had sharp pains, I felt sharp pains when I was forcing her to eat, I just felt a stabbing pain, ah, and then my arm could no longer work. Now how can you help someone else who is helpless when you have also become helpless? [P9, Female, 40]

A fear of contracting HIV was expressed:

Gloves are not available….What do you do? You just wash with your bare hands and end up catching HIV as well and also become ill. [P10, Female, 64]

The above quotes indicate to some extent the sacrificial role of the carers who have taken on burdens beyond their own, for whatever reasons.

#### Care-recipients’ physical/health-related issues

Untrained carers were faced with extreme situations where they simply felt they had to cope. Situations included the following:

…she couldn’t even go to the toilet by herself and I could take her to the toilet, sometimes if I came too late I’d find her messy. [P11, Female, 50]

and:

I used to sleep with her at that time. I would wake up covered with her skin until I said it was better to separate… [P3, Female, 56]

The poor appetite often associated with AIDS seemed to cause a lot of distress probably because of the belief that the antiretrovirals prescribed would not work if the person was not eating well or vomiting:

That is the problem that worries me a lot because if she spends the day without eating…, I can’t …, the pills, the tablets cannot work. [P3, Female, 56]

There were two participants who were caring for patients with mental confusion. Although carers of people with mental impairment face additional challenges, one of these was coping but the other was clearly frustrated:

What he wants to do, some of the things, ah, you can’t get it since he is ill; you don’t know what he wants or doesn’t want. Sometimes you can do something good for him but he can say he doesn’t want it any more, he doesn’t want. [P10, Female, 64]

Additional subthemes were concern for weight loss, deterioration of the patient’s condition and poor mobility, all of which added to the carer’s physical and mental burden.

#### Religious aspects

All but one participant mentioned God during the interviews. Some of the comments seemed to be casual, the ones which many people often make without it necessarily being a true reflection of their religious standing. However, with four participants mentioning God three or more times and one of them mentioning Him seven times, it can be concluded that religion played an important role in the lives of these caregivers.

The supportive role of God in this suffering was expressed:

Only God is helping me and I am truly grateful to him. I would have long died from BP because of thinking too much. [P1, Female, 69]

God is good to me because somehow I find myself with the transport. [P7, Female, 58]

I will be asking God for her to survive. [P2, Female, 44]

This was offset by two other carers who had a more fatalistic approach:

…Nothing can help me to make her well again unless God makes her well. Me there is nothing I can do. [P3, Female, 56]

Ah yes, if God decides to take him, he can take him. What can I do? [P6, Female, 34]

There was also some questioning of God:

I am not lying to you but when I think of all this nearly every day I start to cry, asking God or is it Satan why my daughter is going through all this when some people don’t do so. [P4, Female, 59 years]

Together with this, one caregiver took her responsibility so seriously that a strong element of guilt is apparent:

She is helpless there and you cannot help her as well so what you can do is to cry and ask God for help saying ‘God please help me, what did I do wrong?’ [P8, Female, 58]

#### Family issues

These were matters, which mostly exacerbated the burden of caregiving:

I also have orphans. I have many orphans and the one left by his mother in 2004 is always ill. [P1, Female, 69]

There was a mother struggling with the taboo of washing her adult son because his own wife had abandoned him:

It is difficult indeed because when he first fell ill it was too much for me to touch his body since he is an adult. [P7, Female, 58]

There was a mother caring for her daughter who was very upset with her other children for not supporting her financially because the daughter she was looking after had been her breadwinner:

Actually it is better to say I don’t have any other children to be honest because you can only consider a child who assists you in certain areas. [P4, Female, 59]

In contrast to this another caregiver said:

But anyway, my relatives, my brothers and sisters they help me a lot. Whenever I tell them that I have this problem they come forward and help me. [P9, Female, 40]

Very few people raised the issue of disclosure within the family. One person said:

The problem was telling our children what the pills were for. [P2, Female, 44]

#### Emotional impact

Caregivers freely discussed the emotions they experienced regarding the challenges of the caregiving process. However, they were more reticent about those emotions experienced when witnessing the suffering of their relatives. This may be because of a particular type of resilience or an expectation of family support being non-negotiable within a culture of Ubuntu.

General worry, helplessness and despair were common:

Sometimes I despair so much that tears just flow on their own because I would be feeling so troubled. [P3, Female, 56]

and:

Not having things, you don’t have… Then you worry about you can do about it, that’s troubling that’s troubling, that’s troubling. Where can I go to now? [P10, Female, 64 years]

One of the mothers illustrated the extreme of her despair as follows:

Those who didn’t give birth are better. They don’t see all the things we are seeing. [P10, Female, 64 years]

At the other end of the spectrum of life and death was the hopeless comment:

One day you can end up taking a rope and hanging yourself. [P9, Female, 40]

The pain of seeing loved ones suffering was expressed:

It pains my heart very much because this is my only son. [P7, Female, 58]

Firstly it is not easy, really, to see your daughter’s condition deteriorating day after day. So you develop a stress. Also because you find yourself at times crying. [P11, Female, 50]

Many tears were shed in the interviews themselves.

There was occasionally an element of hope expressed:

Ah I see people who have been very ill surviving. So I have hope that she will survive, maybe as she takes the ARVs she will survive. [P2, Female, 44]

#### Appraisal of the caregiver role

Five caregivers talked about their perspectives on how they viewed their caregiving role. There was a sense of responsibility and obligation, which reflects the resilience and Ubuntu mentioned above:

There is nothing I can do because she is my child… [P3, Female, 56]

Together with this, positive aspects that were vocalised were gratification linked to love for the recipient, a lack of blame and an experience of coping with this role:

But it’s only that it’s difficult but I don’t blame anyone, I don’t blame her. [P8, Female, 58]

I am coping but it is difficult but I am coping, yes. [P11, Female, 50]

…but when it comes to looking after my wife it is hard but I am coping because of all the counselling I have had as you can see. [P5, Male, 36]

## Discussion

The aim of this research was to try and understand the psychosocial impact on carers of HIV and/or AIDS patients through a qualitative process. Both practical and psychological challenges were elicited as were some positive experiences. The themes analysed in the study reflect many similarities with other countries in the world.

The day-to-day struggle for survival amidst poverty and the additional burdens of caregiving with little, if no additional, resources formed the background of most of the stories of caregivers. The prevailing economic hardships of high inflation and lack of employment in Zimbabwe, which were often cited by participants, were a source of great distress for caregivers because of the financial implications for them. All the studies that were reviewed from Africa also revealed that poverty had a major impact on the psychosocial wellbeing of carers.^1,6,7,8,20,21,22^

One mother in this study resorted to selling her household goods to raise money to provide for her ill daughter who previously was the sole breadwinner. This practice was reported in other AIDS caregiver studies.^6,20^ There was a high unemployment rate generally and many caregivers were retired and elderly.^21^ Much of the poverty was compounded by the costs of chronic care including special foods, transport to hospital, washing detergents, etc. As reported in other studies as well,^8,21^ the lack of these distressed caregivers immensely as they felt this compromised the health of the ill.

The health of both carers and patients influenced the psychosocial wellbeing of the carers. Many of the older caregivers had hypertension, diabetes and arthritis, which they felt were being negatively affected by the increased physical role they were expected to play. For example, the limited financial resources did not allow one mother with diabetes to adhere to her diet. Eighty-two per cent of Latina family AIDS carers in the United States also reported chronic conditions such as asthma, hypertension and diabetes.^23^ Tiredness and psychosomatic complaints from the caregivers were common in this and other studies.^1,6,19^ These included headache, abdominal pain, chest pain and anorexia.

Concerns regarding the contracting of HIV were raised in a number of studies.^1,7,22^ In Botswana,^1^ it was found that elderly caregivers did not use the care package they were provided with because they felt that using gloves would imply that they do not love the person in their care. In contrast, caregivers in a Namibian study^7^ expressed anger at the risk they were exposed to. The lack of gloves was also related to specific conditions of patients (e.g. incontinence).

Frustration was expressed regarding patient complaints specifically regarding poor appetite^7,8^ and mental illness,^9^ in common with other studies. Poor mobility and deterioration of the physical condition was distressing to carers. The lack of improvement or worsening of the condition led, in many cases, to feelings of despair in the carers, and these emotions were part of anticipatory grief as found in a US study.^9^

Religious issues similar to those of the study were found in other papers. Fifty-seven per cent of AIDS caregivers in one study^1^ found religion to be a comfort and felt that their faith in God had increased as a result of the illness, while 17% no longer went to church or worshipped at home because of caregiving commitments. Female caregivers in India went through the different stages of questioning God, anger towards Him and finally hope and belief in His compassion. These are similar to emotions found in the study.

Family issues are common themes especially the care of orphans,^1,6,24,25^ but also abandonment^6^ and issues of taboos and disclosure.^26^ These are often linked to emotional aspects as well.

Some patients cried in the interviews while others admitted to crying often because of the difficulties they were encountering to make ends meet. It is not possible from the nature of the study to determine if some of the participants had overt depression, but it is possible considering the stressors they were experiencing, as well as the feelings of emotional pain that they expressed. On the contrary, when asked directly about how they felt about the prognosis of the ill person in their care, most of the participants said that they had hope that the ARVs would help them make a full recovery because they had seen it happen to other people.

Other emotions like anger were found in a number of studies. A comparison between carers of HIV and/or AIDS and dementia patients^2^ found that the former were significantly more anxious and angrier than the latter. Where one of the patients from the study was angry and unappreciative, this distressed her mother greatly. Care dyad conflict has been reported in several studies and causes tensions arising from the mood changes of both partners.^6,7,21^

Despite the challenges that were described by the caregivers some of them still reported that they were coping with the caregiving role. Counselling has helped some of them. Chimwanza and Watkins^8^ postulated that the underplaying of the difficulties of caregiving could be what they termed ‘courtesy bias’ meaning that the participants may have felt that an admission of being troubled by their role as caregivers might be understood as not having enough love for the person they were caring for. This may explain why some of the participants in the study were quick to say they were coping. Some carers indicated a sense of gratification and fulfilment in their roles, which was also found in other studies.^1,2,8^

There is a pervasive underlying theme of duty and the social expectation that relatives will be taken care of under all circumstances by somebody close to them. This ties up with the Ubuntu culture of Africa where the interdependence of all people is a priority.

People’s being is thus determined by this concept of totality by means of which all internal and external dimensions of their existence (spirituality, religion, economy, judicial systems, politics, kinship, language, education, play, art, science, et cetera) are fused into purpose relationships.^27^

This may well be a subconscious driving force in carers who are barely coping with their circumstances.

Although much of the information elicited in the study is not new, it confirms that in Zimbabwe, similar issues confront caregivers as those experienced in the rest of the world. The study also focuses on relevant recommendations that could assist the government with practical interventions such as those set out in the following section. The main thrust of the study is to shift the current focus from patients to include caregivers in a meaningful way.

The following recommendations are made:

- Campaigns by government in conjunction with non-governmental organisations to train and equip health workers to identify caregivers at risk.
- HIV support groups to set up parallel support groups for caregivers.
- Religious groups to identify caregivers in their communities and offer home visits for prayers and other support.
- Social services that provide grants to families with the chronically ill and orphans in their care to be re-established.
- Re-establish effective home-based programmes that support family caregivers. These programmes could utilise humanitarian aid that is being offered to the country to provide services as well as care packages and food parcels.

## Conclusion

The study set out to find out the psychosocial impact of caregiving on family caregivers of chronically ill HIV and/or AIDS patients in Bulawayo, Zimbabwe. There were factors relating to the family, the health and physical aspects of the carers and the care-recipients that compounded the overriding financial burden. The participants experienced a spectrum of emotions from worry, helplessness and hopelessness, despair and sadness to feeling overwhelmed, but some had hope of the recovery of the ill person in their care. Although the sample size was small, the researcher was able to gain a good insight into the experience of these caregivers because of the qualitative nature of the study.

Although some of the interviewees seemed to be at the end of their tether, it is a testament to the resilience of humans that all of them were coping with their burdensome role, with very little social support. The study was carried out in the hope that the findings would contribute towards more concrete support.
